# Thermosonication-induced structural remodeling of faba bean protein: Improved functionality, digestibility, and cholesterol metabolism regulation for cardiovascular health^☆^

**DOI:** 10.1016/j.ultsonch.2026.108038

**Published:** 2026-09-04

**Authors:** Jawad Ashraf, Muawuz Ijaz, Zeeshan Ali, Sanabil Yaqoob, Miao Bai, Hafiz Umer Javeed, Ayhsa Imtiaz, Sumei Zhou, Qing Shen, Ting Chen

**Affiliations:** a College of Food Science and Engineering, Bohai University, Jinzhou, Liaoning 121013, China; b Department of Animal Sciences, CVAS-Jhang 35200, University of Veterinary and Animal Sciences, Lahore 54000, Pakistan; c Laboratory of Food Nutrition and Clinical Research, Institute of Seafood, Zhejiang Gongshang University, Hangzhou 310012, China; d Guangxi Zhuang Autonomous Region Engineering Research Centre of Marine Food Nutrition and Processing Technology Innovation, Guangxi College and University Key Laboratory of High-Value Utilization of Seafood and Prepared Food in Beibu Gulf, College of Food Engineering, Beibu Gulf University, Qinzhou 531011 China; e School of Fisheries and Life Sciences, Shanghai Ocean University, Shanghai China; f School of Food and Health, Beijing Technology and Business University, Beijing 100048, China; g Department of Cardiology, First Affiliated Hospital, Zhejiang University School of Medicine, Hangzhou 310003, China

**Keywords:** Faba bean protein isolate, Thermosonication, Structure-function relationship, Cholesterol metabolism, HMG-CoA reductase, Functional foods

## Abstract

Faba bean protein isolate (FPI) is a promising plant-based protein ingredient; however, its compact molecular structure restricts its functional performance and bioactive potential. The effects of heat treatment (H-FPI), ultrasonication (U-FPI), and sequential thermosonication (UH-FPI) on the structural characteristics, techno-functional properties, and hypocholesterolemic activity of FPI were investigated in vitro and in vivo. Conformational changes of the protein matrix induced by ultrasound and thermosonication cause a decrease in the surface hydrophobicity, an increase in protein digestibility, and an improvement in functional properties over a wide range of pH. The in-vitro studies revealed that structural modification was also effective in improving the biological activity; in particular, the degree of hydrolysis was enhanced from 8.02 ± 2.62% (FPI) to 21.72 ± 2.25% (UH-FPI), while the micellar inhibition of cholesterol was enhanced from 23.4 ± 1.13% (FPI) to 48.5 ± 0.63% (UH-FPI) and higher HMG-CoA reductase inhibitory activity from 84.17 ± 2.65% (FPI) to 58.79 ± 3.52% (UH-FPI) indicating its potential in inhibiting intestinal cholesterol absorption and cholesterol biosynthesis. Furthermore, in vivo studies using high-fat (HF) diet-fed hamsters showed that UH-FPI improved the serum lipid profile and cholesterol metabolism-related proteins. The expression analyses further confirmed that UH-FPI upregulated the expression of cholesterol efflux-related proteins (ABCG5 and ABCG8), hepatic cholesterol metabolism regulators (LXR-α, CYP7A1, and LDL-R), and downregulated the expression of the intestinal cholesterol uptake-related protein NPC1L1 upon normalization with the HF group. The results indicate that thermosonication could be used to alter faba bean protein’s structure and modulate cholesterol metabolism, leading to improved functional and hypocholesterolemic properties, making it a suitable functional food ingredient for the promotion of cardiovascular health.

## Graphical abstract

**Figure:**
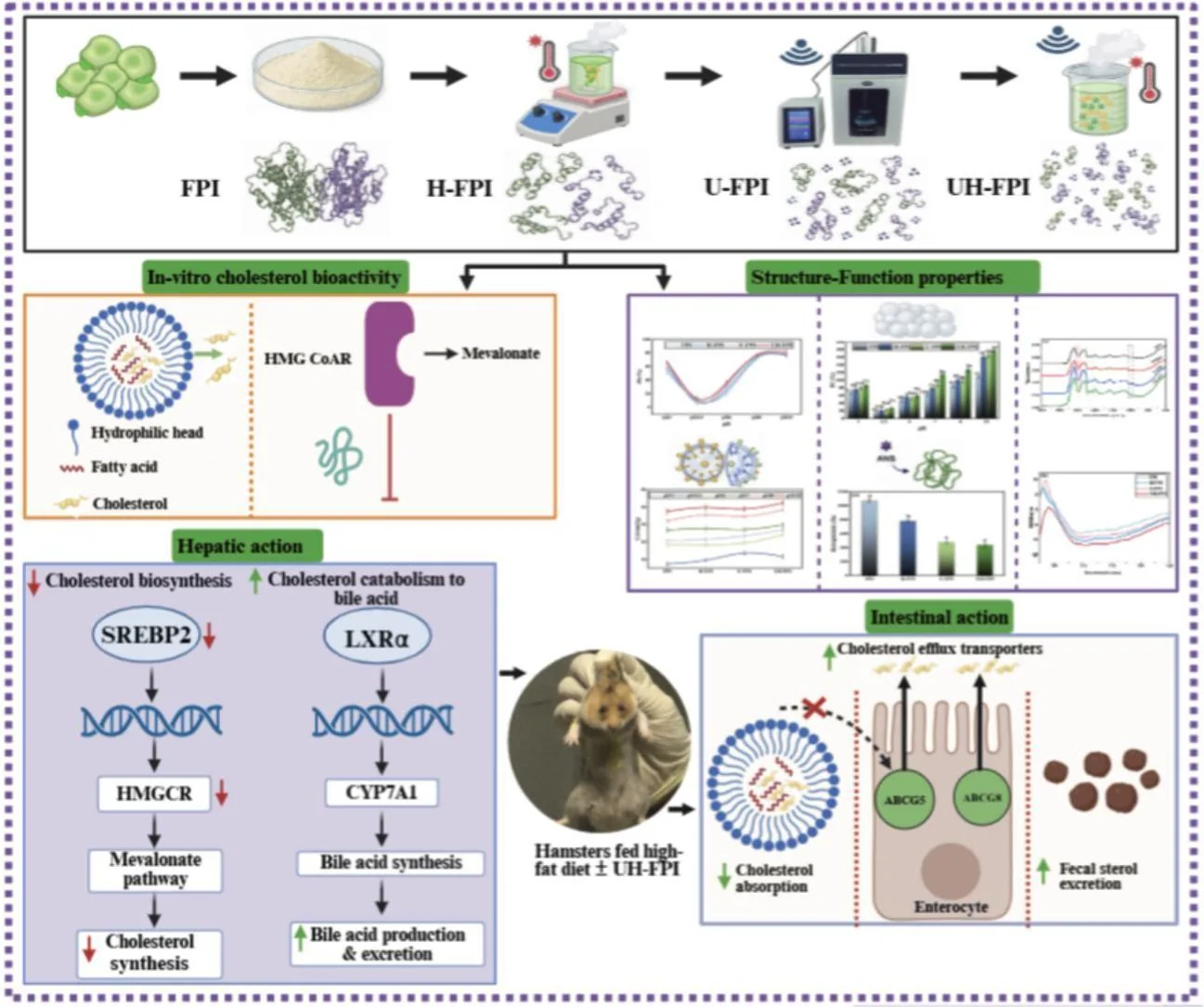

## 1 Introduction

Social awareness and improvements in living standards over the past decade, driven by economic growth, have led to a shift in food preferences toward nutritious and palatable foods. As a result, the dietary supplement market has grown rapidly, with annual revenue surpassing $30 billion [1]. Meanwhile, global protein demand has also risen, leading to increased interest in plant proteins owing to their protein quality and essential amino acid contents [2]. Plant proteins have physicochemical properties, aside from their nutritional aspects, that affect food processing, storage stability, texture and sensory quality. This is a continuous “protein transition” that involves a gradual shift towards plant-based proteins as part of sustainable and healthy diets [3]. Among the plant proteins, faba bean is an excellent protein source with a high protein density, neutral amino acid composition, and relatively low cultivation demands [4]. The proteins found in it are primarily globulins, such as vicilin and legumin, whose nutritional and functional properties are most important. The storage proteins of faba beans are globulins, which can be separated into 7S vicilin-type and 11S legumin-type classes based on sedimentation coefficients, and contribute 70–80% of the total protein. Albumins, though in lesser quantities, are also rich in amino acids containing the sulfur element and thus are nutritionally better than many other seed proteins [5]. In the protein fraction, there are differences in the structures that result in different characteristics of solubility, thermal stability, and techno-functional properties [5]. Faba bean proteins and peptides have attracted attention due to their antioxidant properties and other health-promoting properties [6], in addition to their use as sustainable protein ingredients.

However, due to its natural structure, faba bean protein has limited applications in food systems. Like other legume proteins, the faba bean proteins have tightly folded globular structures, where hydrophobic interactions, hydrogen bonds and disulfide bonds play a key role in stabilizing the structures. These small structures limit the ability of the protein to interact with water, make it less susceptible to enzymes, and decrease the availability of functional and biologically active amino acid residues [7]. The low solubility and techno-functional performance of native proteins from faba beans in complex food matrices is therefore common. Thus, significant efforts have been devoted to physical modification methods that can cause controlled conformational changes that can enhance functional and biological properties.

Given the growing and growing problems of hypercholesterolemia and cardiovascular diseases, there has been a renewed interest in functional foods that can naturally modulate lipid metabolism. Cholesterol homeostasis is achieved through a coordinated regulation of intestinal absorption, endogenous biosynthesis, hepatic uptake, and conversion to bile acids. At the intestinal level, NPC1L1 (Niemann–Pick C1-like 1) is a key cholesterol uptake transporter of enterocytes, whereas ABCG5/ABCG8 (ATP-binding cassette G5/G8) facilitates cholesterol and sterol elimination by promoting their efflux from enterocytes into the intestinal lumen and from hepatocytes into bile. The de novo synthesis of cholesterol in the liver is mainly controlled by SREBP2 (sterol regulatory element-binding protein 2), which regulates the expression of cholesterol biosynthetic genes, including HMGCR (3-hydroxy-3-methylglutaryl-CoA reductase), the rate-limiting enzyme in cholesterol synthesis. LDL-R (Low-density lipoprotein receptor) also promotes the uptake of LDL particles from the circulation in the liver and thus plays a significant role in plasma cholesterol clearance [8]. Furthermore, CYP7A1 (cholesterol 7α-hydroxylase) catalyzes the rate-limiting step in the classical pathway of bile acid synthesis, providing an important route for hepatic cholesterol catabolism and elimination. LXR-α (Liver X receptor-α) is a sterol-sensitive transcription factor that favors cholesterol disposal and has the potential to modulate cholesterol efflux and bile acid metabolism genes [9]. Other proteins, such as ACAT2 (acyl-CoA:cholesterol acyltransferase 2) and MTP (microsomal triglyceride transfer protein), have been involved in intestinal cholesterol esterification and lipoprotein assembly, respectively [10]. Therefore, the modulation of these interrelated proteins can affect cholesterol absorption, hepatic production, transport and elimination, thus providing a mechanistic approach to the assessment of the hypocholesterolemic activity of bioactive food proteins [11].

Previous studies have demonstrated that ultrasound can effectively modify the structural and functional properties of faba bean protein isolate (FPI). It has been reported that high-intensity ultrasound can cause changes in the secondary structure of FPI, resulting in the improvement of its solubility, interfacial properties, and foaming performance. More recent studies also revealed that the hydrophobicity, particle size, thermal properties, and solubility of FPI were also modified after ultrasound treatment, depending on ultrasound frequency and treatment conditions [12]. It can be hypothesized that the simultaneous application of heat and ultrasound could be more efficient, as thermal treatment promotes protein unfolding, whilst ultrasound would disperse aggregates and enhance the dispersion of molecules, making them more accessible for hydrolysis [13]. Thermal denaturation may reveal hydrophobic residues and reactive amino acid groups that may increase solubility, emulsification, digestibility and intermolecular interactions. However, excessive heating may also facilitate aggregation by way of hydrophobic associations and sulfhydryl–disulfide interchange reactions, which may decrease functionality [13]. Thermosonication has also recently been explored for FPI, where improved solubility, particle size, emulsification, and viscoelastic properties were observed compared to conventional heating [7]. Most of these studies, however, are centered on structural, physicochemical, and techno-functional properties. However, the impact of thermosonication-induced structural remodeling on the biological activity of FPI remains poorly investigated. Specifically, the limited information available concerning the digestibility and the hypocholesterolemic potential of FPI and the relationship between structural changes induced by thermosonication and the cholesterol regulation mechanisms [14]. Hence, there is a need for a systematic study that combines structural characterization, techno-functional properties, digestibility, in vitro cholesterol-related biological activity, and in vivo validation. To the best of our knowledge, there is a scarcity of information about the synergistic impacts of heat treatment and ultrasound on the structural–functional properties and hypocholesterolaemic activity of faba bean protein. To overcome these limitations, the present study systematically explored the relationship between thermosonication-induced structural remodeling, hydrolysis and hypocholesterolemic activity of FPI, in contrast to previous studies that mainly focused on the physicochemical and techno-functional attributes of ultrasound or thermosonication treatment. Moreover, in vitro assays and a hamster model fed a high-fat diet were used to assess the potential cholesterol-lowering mechanisms of thermosonicated FPI. This integrated approach provides new insight into the structure–function–bioactivity relationship of FPI, as well as the potential use of thermosonication as a promising approach for producing plant protein ingredients with increased nutritional and cardiovascular-health-related functionality.

## 2 Materials and methods

### 2.1 Materials

Faba beans were sourced from major legume-producing regions in China (Hebei and Xinghai provinces) and kept at 4 °C until further utilization. The BCA (bicinchoninic acid) protein assay kit, total cholesterol assay kit, ≥250 U/mg pepsin and ≥ 250 U/mg trypsin were procured from Solarbio, China, while ANS (1-anilino-8-naphthalene sulfonate) and the CS1090 HMG-CoA reductase assay kit were obtained from Sigma-Aldrich, USA. All additional compounds were at standard analytical levels. Faba beans (free from dust and foreign materials) were milled using a FOSS Cyclotec™ 293 mill and passed through an 80-mesh film to obtain uniform flour.

### 2.2 Preparation of faba bean protein isolate (FPI)

The FPI was isolated from the faba bean using the method developed by [15] with slight alterations. In short, flour was mixed in distilled water (1:10, w/v) with continuous stirring to get a homogeneous suspension. To encourage protein solubilization, the pH was set at 8.5 (1 M NaOH), and the mixture was maintained at 40 °C for 1 h. The supernatant was collected by eliminating insoluble fractions by centrifugation at 4500 × g for 25 min. Precipitation of protein was induced by lowering the pH to 4.5 (with 1 M HCl) and equilibration for 1 h at room temperature. The precipitated protein was obtained by centrifugation under the above-mentioned conditions, rinsed with distilled water until neutral pH following the freeze dried and the protein powder was kept at 4 °C until further utilization.

### 2.3 Ultrasound and thermal treatments

Dispersions of protein (5%, w/v) were made in distilled water and hydrated for 2 h at room temperature with magnetic stirring. The 20–25 kHz, 950 W power Scientz-IID sonicator (Ningbo Xinzhi Biotechnology Co., Ltd, China) equipped with a titanium alloy probe of 6 mm was used for ultrasound processing. A working volume of 150 mL was sonicated at 10% amplitude with a 1.5 cm probe immersion depth. The system was operated in pulsed mode (2 s on / 2 s off) for 10 min to modulate cavitation dynamics and to reduce bulk heating. A temperature-controlled ice-water bath was employed to dissipate excess thermal energy and maintain quasi-isothermal conditions during acoustic cavitation. Comparative thermal treatment was performed by conventional heating of protein dispersions in boiling water for 10 min, which allows only thermally driven structural transitions in FPI. The samples were immediately cooled with an ice bath. In addition, UH-FPI was produced by applying a sequential thermosonication method, where the FPI dispersion was first heated for 10 min in boiling water and then ultrasound treated for another 10 min under a pulsed mode (2 s on/2 s off). The sequential treatment method was used in the study of the synergistic effect of thermal unfolding and ultrasound-induced cavitation on structural rearrangement and functional properties of FPI.

### 2.4 Acoustic energy Determination

The acoustic energy input was measured calorimetrically by measuring the temperature rise of the medium and then using the assumption of complete conversion of the ultrasonic energy into heat under the experimental conditions to convert this energy into mechanical energy. Acoustic power (P) was derived from the initial linear region of the temperature–time profile (600 s) according to:

P = m × CP × (dT/dt)

In which m is the mass of the treated medium (g); CP is the specific heat capacity of water (4.18 J g-1 K-1); dT/dt is the initial temperature increase rate (K s-1). The volumetric acoustic intensity was expressed as power density (W cm-3), representing the effective energy distribution within the sonicated solution. The acoustic intensity measured under the applied conditions was 10.45 ± 0.77 W cm^–3^.

### 2.5 Structural properties

#### 2.5.1 Fourier transform infrared (FTIR) spectroscopy

FTIR (Fourier Transform Infrared) spectroscopy was applied to analyze the tertiary conformational characteristics of FPI, H-FPI, U-FPI, and UH-FPI following the procedure reported by [16] with a spectral range of 600–2000 cm^–1^ and a resolution of 4 cm^–1^. FTIR spectra of FPI and treated FPI samples were recorded on a Fourier-transform infrared spectrophotometer (Bruker, Bremen, Germany) attenuated with a total reflection system (MKII Golden Gate; Specac, Orpington, UK). The spectral data obtained were processed and interpreted by means of the OPUS software (version 6.5), which is integrated with the instrument system.

#### 2.5.2 Circular dichroism (CD) spectroscopy

The secondary structural characteristics of FPI, H-FPI, U-FPI, and UH-FPI were studied with a circular dichroism (CD) spectropolarimeter (MOS-450, Biologic Science Instruments, Grenoble, France) according to the method of [17]. Freshly prepared protein dispersions (0.25 mg mL^–1^) were used and examined in the far-UV region of 190 to 240 nm. The measurements were performed with a quartz cuvette with a 0.1 cm path length at a 1000 nm/min scanning rate, 1 nm spectral interval and 0.5 s response time. The reference solution was phosphate buffer (0.01 mM) (pH 7.4), and the baseline correction was performed. All spectra were made in duplicate, and the information was evaluated using Bio-Kine software integrated with the instrument system.

### 2.6 Surface hydrophobicity (Ho)

The surface hydrophobicity of FPI was measured using the fluorescence of 1-anilino-8-naphthalene sulfonate (ANS) fluorescent method with a few modifications as described by [17]. Different concentrations of FPI (0.2–1.0 mg/mL) were dispersed in 20 μL of ANS solution (8 mM) and then incubated in the dark for 30 min at room temperature. The fluorescence intensity of ANS-bound protein samples was determined at 380 nm excitation and 470 nm emission wavelength with a fluorescence spectrophotometer. The initial slope of fluorescence intensity versus protein concentration was calculated by linear regression and used as the surface hydrophobicity index.

### 2.7 Functional properties

#### 2.7.1 Solubility

The solubility of FPI was explored by following [18], with slight modifications. Protein dispersions (10 mg/mL) were prepared in deionized water, and the pH of the dispersions was adjusted to 3.0, 4.5, 6.0, 8.0 and 10.0 with HCl (1 N) or NaOH (1 N). The suspensions were then magnetically stirred for 3 h at 25 °C, following complete hydration and equilibration of the suspension. The specimens were then centrifuged at 8000 × g for 10 min, and soluble protein content in the supernatant was assessed by bicinchoninic acid (BCA) assay.

#### 2.7.2 Foaming and emulsifying properties

The foaming and emulsifying property of FPI was measured according to the method of [19] with some modifications. In brief, the FPI dispersions were transferred to a graduated glass cylinder and homogenized using an IKA T18 digital ULTRA-TURRAX homogenizer (Staufen, Germany) at 12,000 rpm for 3 min to obtain foam. The FC (foaming capacity) and FS (foaming stability) were identified by calculating the amount of foam volume increased immediately after homogenization, and stability was calculated after a 20-minute time interval. FC and FS were calculated using the following equations:

FC (\%) =VFVI×100

FS (\%) =VfVI×100

For emulsifying properties, the FPI solution (5 mL) was added to soybean oil (5 mL) and homogenized at 12,000 rpm for 2 min. The emulsion was freshly prepared and vortexed for 10 s after 100-fold dilution in 0.1% SDS solution. The absorbance was measured immediately after homogenizing for the emulsifying activity index (EAI) and 10 min after homogenizing for the emulsion stability index (ESI). The EAI and ESI were determined using the following equations:

EAI (m2g) =2×2.303×AO×DilutionC×0.0001×ϕ×1000

$$\text{ESI (min)}=\frac{\text{A10}\;\times {\text{A}}_{\text{o}}}{{\text{A}}_{\text{o}}\;\text{- A10}}$$

where A_o_ and A10 are the spectrophotometric absorbance at 0 min and 10 min, respectively, and C is the solution concentration (*ɸ*=0.25)

### 2.8 In vitro activities

#### 2.8.1 Simulated gastrointestinal digestion

The in vitro gastrointestinal digestion of FPI was performed by following our previous method [17]. FPI dispersions (5%, w/v) were acidified to pH 2.0 with 1 M HCl, and then pepsin was added at 4% (w/w, protein basis), and the solution was then stored for 60 min at 37 °C in a thermostatically controlled water bath to represent gastric digestion. The pH was then raised to 5.3 by adding 0.9 M NaHCO_3_ and then to pH 7.0 by adding 1 M NaOH before the addition of trypsin (4%, w/w). The samples were then digested in the intestinal mimicry at 37 °C for 120 min. After that, the enzymatic activity was impeded by putting the mixture in boiling water for 12 min. The digested suspensions were then centrifuged (14,000 × g, 8 min), and the supernatants were processed for more examination.

#### 2.8.2 Degree of hydrolysis

The degree of hydrolysis (DH) was calculated using the FPI solubility in trichloroacetic acid (TCA), as described by [15]. In brief, 10 mL of digested hydrolysate was suspended with 10% TCA solution and incubated for 15 min to precipitate the protein that had not been digested. solutions were then centrifuged at 12,000 × g for 15 min, and the nitrogen concentration in the soluble fraction was measured by the Kjeldahl method. The DH was determined based on the following equation:

$$DH\left(\%\right)\;=\;\frac{Hydrolysate\;Protein}{Total\;Protein}\;\times \;100$$

#### 2.8.3 In vitro cholesterol micellar solubility

The cholesterol micellar solubility assay was carried out as [17]. Micellar solutions (pH 7.4) consisting of sodium taurocholate (10 mM), cholesterol (2 mM), oleic acid (5 mM), NaCl (132 mM), and sodium phosphate buffer (15 mM) were homogenized by ultrasonication (400 W, 20 kHz) for 20 min. FPI samples were then introduced to the micellar system at a concentration of 2 mg/mL, followed by storage at 37 °C for 24 h. The mixtures were then stored, followed by centrifugation at 10000 × g for 35 min, after which the cholesterol concentration in the supernatant was evaluated using a total cholesterol assay kit. To determine the inhibitory effect on micellar cholesterol solubility, the following equation was used:

$$Inhibition\;Activity\left(\%\right)=\;\frac{Co-Cs}{Co}\times 100$$

where Co denotes the cholesterol concentration in the micellar solution, and Cs represents the cholesterol concentration in the presence of FPI samples.

#### 2.8.4 HMG-CoA reductase inhibitory activity

The inhibition of HMG-CoA reductase was assessed by employing a commercial assay kit obtained from Sigma-Aldrich (CS10190). The assay is based on monitoring the NADPH oxidation catalyzed by HMG-CoA reductase, while pravastatin served as the positive control inhibitor. Sample preparation and reagent handling were carried out in accordance with the instructions of the manufacturer. The reaction mixture contained either 1 µL of pravastatin or FPI reagent at a concentration of 50 µg/mL. The changes in absorbance associated with NADPH consumption were monitored using a FlexStation 3 microplate reader (Molecular Devices, USA). Kinetic measurements were recorded over a period of 600 s with readings collected at 20 s intervals.

### 2.9 DPPH radical scavenging activity

The free radical scavenging ability of the hydrolysates was assessed using the DPPH assay following the procedure reported by [17]. with slight modifications. Briefly, 2 mL of 0.1 mM DPPH methanolic reagent was combined with 2 mL of the sample extract prepared at various concentrations. The mixtures were vortex-mixed properly and stored in the dark at ambient temperature for 30 min. After storage, the absorbance was determined at 517 nm by a microplate reader (SP-Max 2100). Butylated hydroxytoluene (BHT) was used as the reference antioxidant. Radical scavenging activity was calculated according to the following equation:

$$\text{Scavenging Activity}\;\left(\%\right)=\left[\text{1-}\frac{A_{1}\text{-}{\text{A}}_{2}}{A_{0}}\right]\;\text{× 100}$$

where A0 represents the absorbance of the DPPH control solution, A1 corresponds to the absorbance of the sample containing DPPH solution, and A2 is the absorbance of the blank.

### 2.10 Effect of UH-FPI in hypercholesterolemic hamsters

After optimization, UH-FPI was chosen for the in vivo study as it showed the strongest cholesterol-lowering effect during the in vitro experiments. Compared with heat-treated or ultrasonicated samples alone, thermosonicated (UH-FPI) produced a greater reduction in micellar cholesterol synthesis and exhibited the highest inhibitory effect against HMG-CoA reductase activity. These results indicate that the combined ultrasound and heat treatment was more effective in improving the hypocholesterolemic properties of FPI.

#### 2.10.1 Animals and experimental diets

All animal-related experiments were conducted in accordance with the instructions issued by the Ethical Committee of the Experimental Animal Center, Institute of Medicinal Plant Development, Chinese Academy of Medical Sciences and Peking Union Medical College under the approval number SLXD-20250513007. Male hamsters (89.51 ± 0.57 g) were brought from Beijing Vital River Laboratory Animal Technology Co., Ltd. Under SPF (specific pathogen-free) conditions, the animals were acclimatized at 20.0 ± 0.5 °C, 50 ± 10% relative humidity and a controlled 12 h light/dark photoperiod with unrestricted access to water and food.

Experimental diets were formulated in accordance with the recommendations of the AIN-93G diet with some minor modifications (Table S1).

During the study period, food consumption was monitored on a daily basis to ensure the same level of protein, lipid and starch intake in all treatment groups, and the body weight was recorded weekly. Hamsters were fasted overnight prior to sample collection at the completion of the experiment. Under diethyl ether anesthesia, blood samples were drawn from the orbital venous plexus and then centrifuged at 3500 rpm for 10 min at 4 °C to isolate serum. The liver and intestine were obtained and frozen in liquid nitrogen and kept at − 80 °C for further investigation. The effects of the dietary treatments on the physiology of the hamsters were determined by recording body weight changes weekly during the feeding trial.

#### 2.10.2 Serum biochemical analysis

The serum lipid parameters such as TC (total cholesterol), TG (triglycerides), HDL-C (high-density lipoprotein cholesterol) and LDL-C (low-density lipoprotein cholesterol) were determined by a biochemical analyzer with complete automation (Hitachi, Tokyo, Japan).

#### 2.10.3 Determination of liver and intestinal lipid metabolism biomarkers

Commercially available ELISA (enzyme-linked immunosorbent assay) kits were used to measure hepatic biomarkers related to lipid metabolism, such as HMGR (3-hydroxy-3-methylglutaryl coenzyme A reductase), LXR-α (liver X receptor alpha), CYP7A1 (cholesterol 7 alpha-hydroxylase), LDL-R (low-density lipoprotein receptor), and SREBP2 (sterol regulatory element-binding protein 2), following the instructions of the manufacturer. Likewise, the levels of intestinal lipid transporting proteins (ATP-binding cassette transporters G5 and G8 (ABCG5 and ABCG8), Niemann–Pick C1-like 1 (NPC1L1), acetyl-CoA acetyltransferase 2 (ACAT2), and MTP (microsomal triglyceride transfer protein) were calculated using the corresponding ELISA kits in accordance with the instructions of the manufacturer.

In brief, tissue homogenates were prepared in ice-cold physiological saline and centrifuged at 4 °C to get the supernatant. The supernatants were collected for measurement of the biomarkers. Absorbance was quantified using a microplate reader, and concentrations of the biomarkers were determined from the standard calibration curve supplied with each of the assays. Results obtained were reported in ng/mL, pg/mL, or mmol/L as per the biomarker. Analysis was carried out by a commercial biotechnology service provider using standard analytical processes.

### 2.11 Statistical analysis

The statistical analysis was done using 21.0 SPSS software (SPSS Inc., Chicago, IL, USA). Information was analyzed by one-way ANOVA (analysis of variance), and significant differences in treatment means were determined by Duncan's multiple range test at the 5% significance level. Pearson correlation was also used to examine the relationship among the variables measured. Each experiment was repeated thrice, and the findings are shown in the form of mean ± SD.

## 3 Results and Discussion

### 3.1 Faba protein isolates (FPI)

Faba bean protein was isolated by isoelectric precipitation, and the proximate analysis and amino acid composition of FPI are depicted in Table S2. The purity of protein isolates was 90.58 ± 0.48%. Whereas, moisture, ash, fat, and carbohydrate contents were 2.57 ± 0.09%, 4.33 ± 0.14%, 0.85 ± 0.26% and 2.3.0 ± 0.13%, respectively. Protein content of FPI is comparable with the earlier reported results [15], [19] for faba bean protein isolates (89.76 ± 1%) that fulfilled the commercial need of protein isolates, i.e., 90%. Furthermore, the composition of protein is presented in Table S3.

### 3.2 Structural modification

#### 3.2.1 FTIR spectroscopy

**Fig. 1:**
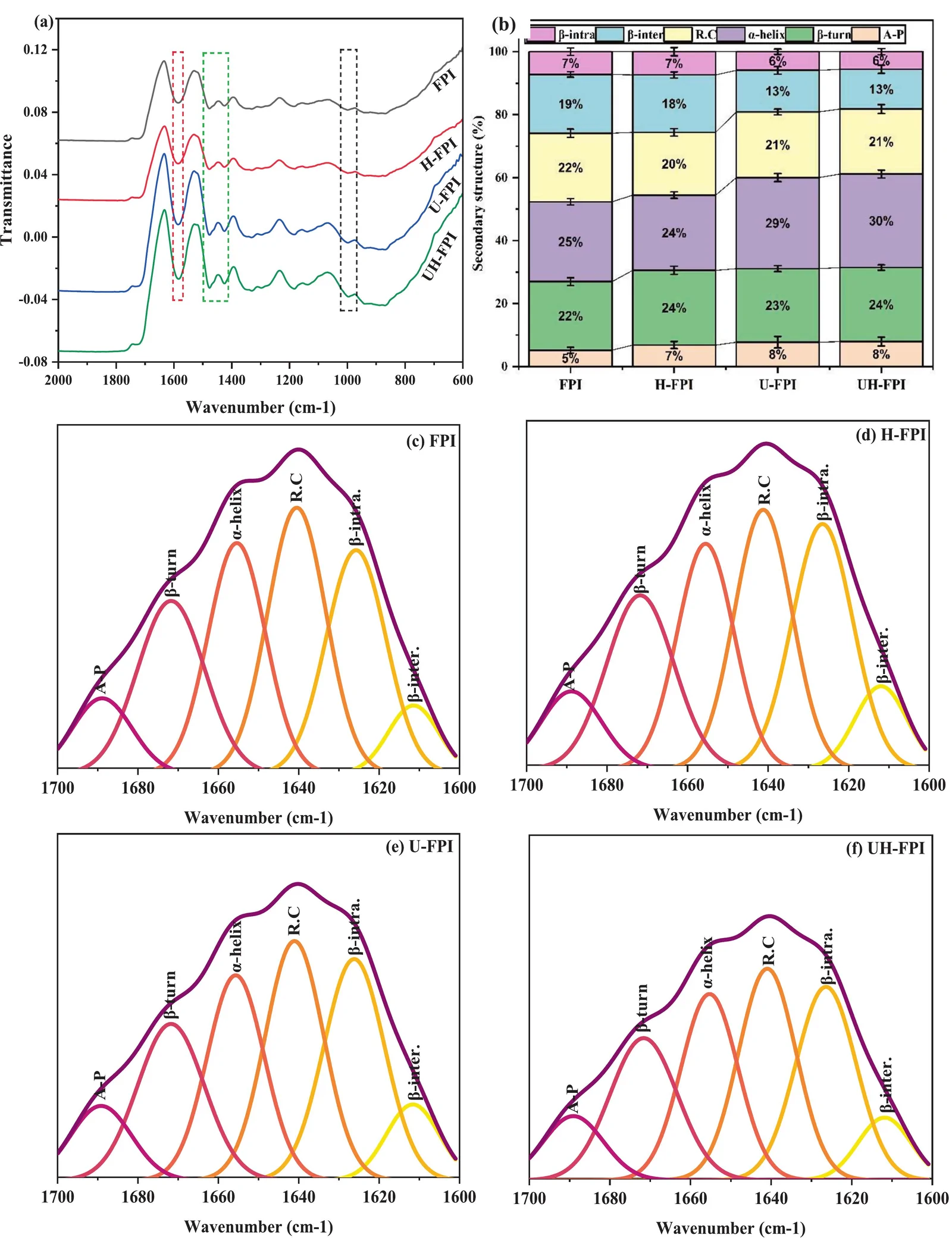
FTIR spectra (a); relative percentage of secondary structural contents (b) and deconvolution peaks (c-f) of FPI, H-FPI, U-FPI and UH-FPI.

The FTIR spectra of FPI, H-FPI, U-FPI, and UH-FPI are shown in Fig. 1 (a). All the spectra had the typical protein absorption bands, confirming that the heat, ultrasound, and thermosonication treatments had modified the protein conformation without modifying its basic chemical structure. The amide I band (1600–1700 cm-1) is mainly attributed to the C=O stretching and is very delicate for the protein’s secondary structure. In treated samples, peak intensities and peak shapes were observed to be different to that of the native FPI, especially in the case of UH-FPI. This indicates a change in hydrogen bonding and in secondary structure (α-helices, β-sheets) of the protein. In faba bean protein, similar observations were made, where the cavitation effect caused protein unfolding and reorganization in its structure during ultrasound treatment [20].

The amide II region (∼1540 cm^–1^) that represents C–N stretching vibrations and N–H bending also showed changes. The lower intensity of the U-FPI and UH-FPI signals suggests the disruption of intermolecular interactions and exposure of formerly buried peptide groups. It has been observed that the increased spectral modifications of UH-FPI compared to H-FPI and U-FPI indicate an enhancement effect of the heat and ultrasound on the denaturation and flexibility of the protein molecules [18]. In addition, the differences in the amide III region (1400–1450 cm-1) and fingerprint region (950–1050 cm-1) are indicative of changes in the protein conformation and non-covalent interactions. The more diffuse peaks seen in UH-FPI suggest a less organized structure and increased mobility of the molecules. The results are consistent with the earlier studies reporting that thermosonication breaks up protein aggregates, reduces hydrogen bonding, and induces conformational changes in legume proteins [21].

The secondary structure of FPI subjected to different processing conditions was characterized using Origin 2021 by data transformation and deconvolution of the amide I region. The relative percentage and deconvolution of the amide I region showed that the secondary structure of FPI was significantly changed by structural modification, as illustrated in Fig. 1 (b-f). The random coil fraction (22–20%) and the β-intermolecular sheet (19–18%) decreased slightly, while the β-turn (22–24%) and antiparallel β-sheet (5–7%) contents increased in H-FPI when compared to the native protein. In the case of U-FPI and UH-FPI, more significant conformational changes were observed, with a significant rise in α-helix content (25–30%) and antiparallel β-sheet structures (8%) and a decrease in β-intermolecular sheet (13%) and random coils (21%). The results suggest that thermosonication caused the disruption and reorganization of non-covalent interactions, leading to the protein refolding into a more ordered and compact conformation, contributing to the improvement of functional and bioactive attributes of the modified FPI.

Overall, the UH-FPI spectra showed the largest spectral changes, indicating that thermosonication was more effective than heat or ultrasound in unfolding the structure. The conformational changes may account for the increased solubility, surface hydrophobicity, digestibility, and cholesterol-lowering activity of UH-FPI, as these changes rendered the functional and enzymatically susceptible sites of the protein more accessible [7].

#### 3.2.2 Circular dichroism (CD)

**Fig. 2:**
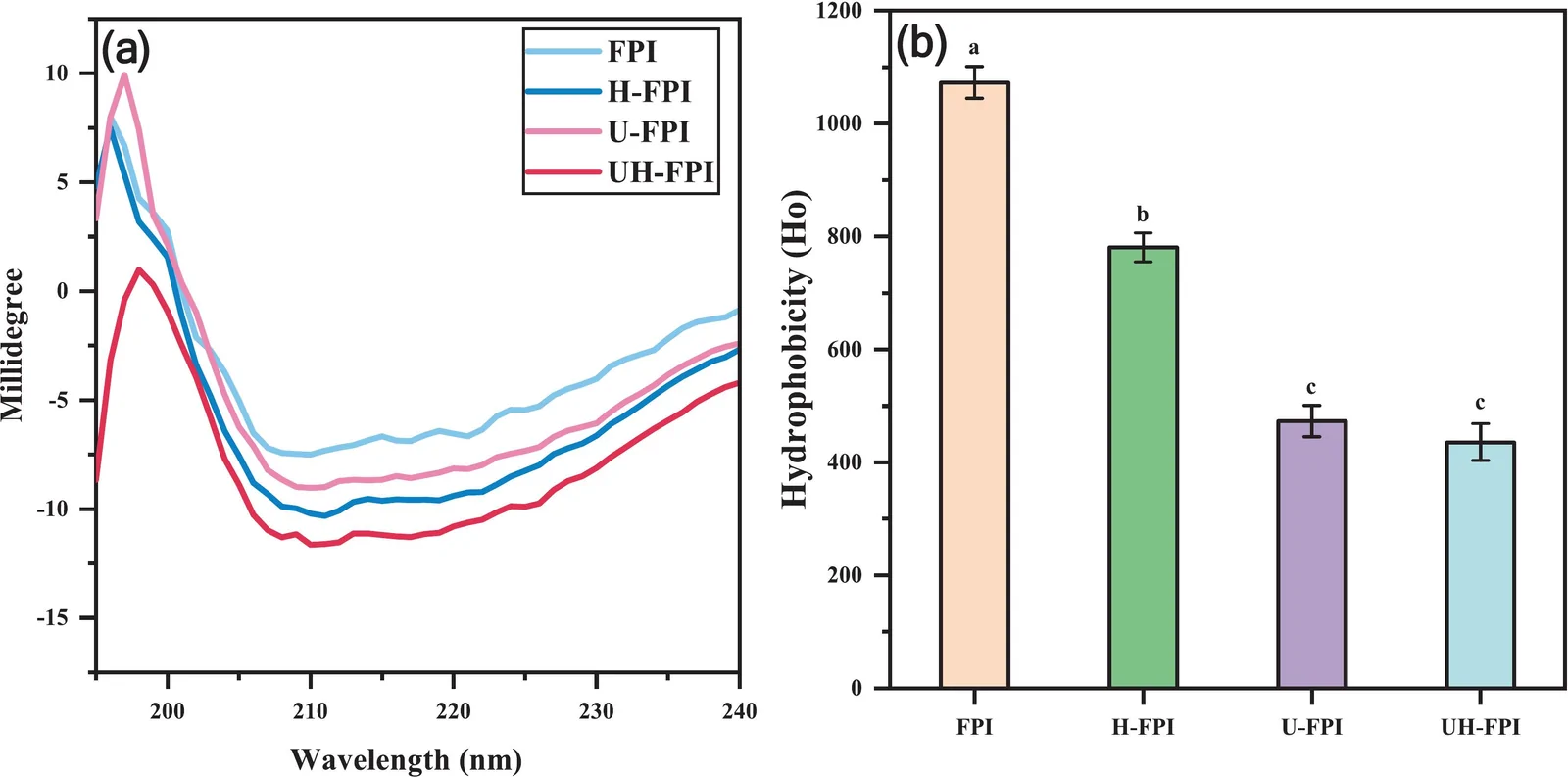
CD spectra (a) and surface hydrophobicity (b) of FPI, H-FPI, U-FPI and UH-FPI.

The far-UV CD spectra in Fig. 2 (a) exhibited significant modifications in the protein’s secondary structure after heat, ultrasound, and combined treatment. The H-FPI exhibited the typical α-helical structure, with two negative ellipticities at 208 and 222 nm. The protein's thermal stability is reduced upon heating to high temperatures, but, as other studies have reported, moderate heating can stabilize or slightly increase the amount of α-helices in the protein before its further thermal denaturation [22]. This indicates that the heating conditions applied in the present study largely did not affect the native conformational arrangement of the protein. By contrast, U-FPI resulted in a wide negative peak around 217 nm and a significant decrease in the 222 nm signal, suggesting a shift towards β-sheet-rich structures, along with a loss of α-helical structure. Partial unfolding or partially unfolded (molten globule) structures have also been reported in ultrasonically treated proteins, in which intramolecular hydrogen bonding is damaged by cavitation and by the shear stresses, leading to the formation of partially unfolded or molten globule structures [23], [24]. The results from earlier studies showed that the secondary structure of proteins is progressively destabilized by ultrasound. The UH-FPI spectrum exhibited an intermediate structural pattern, that is, a less intense minimum at 208 nm than for H-FPI, but a more significant region of the spectrum at 215–225 nm than was seen for U-FPI. It was found that the effects of heat and ultrasound were not strictly additive. Treatment with ultrasound seems to disrupt ordered helical domains, while further thermal treatment seems to bring about the partial reorientation or refolding of specific segments, leading to an intermediate conformation [17], [23]. The destabilization of α-helical structures is confirmed by the progressive decrease of the intensity of the 222 nm band. All of these show that each processing method produced a unique conformational profile: heat treatment largely preserved helical structure, ultrasound resulted in greater β-sheet content and some unfolding, and the combination of the two resulted in a structure found to be more in an intermediate state between the native and molten globule.

### 3.3 Surface hydrophobicity (Ho)

As shown in Fig. 2 (b), thermal and ultrasonic treatments had a significant effect on the surface hydrophobicity (H_0_) of FPI. The protein was exposed to a higher number of hydrophobic residues on the surface as seen in the untreated sample (FPI), followed by the H-FPI, U-FPI and UH-FPI samples. ANS fluorescence-based hydrophobicity of proteins is closely correlated with conformational organization, intermolecular interactions and aggregation properties. The moderate decrease seen in H-FPI is indicative of partial unfolding of the structure followed by rearrangement into a more compact structure, which limits the accessibility of non-polar amino acid residues. Such decreases in hydrophobic exposure in plant and dairy proteins have been observed after controlled thermal processing, which results in molecular ordering and conformational stabilization [25].

The significantly smaller values of H_0_ for U-FPI and UH-FPI suggest that ultrasound treatment led to greater structural changes. With the help of high-intensity ultrasonication, cavitation, localized shear stress, and transient high-energy microenvironments are produced, which lead to the disruption of native protein conformations. Unfolding usually enhances the exposure of hydrophobic surface area, but if this is left for too long, intermolecular hydrophobic interactions and protein aggregation can lead to a reduction in the exposure of hydrophobic surface area. These hydrophobic changes, similar to those described for ultrasonically modified legume and myofibrillar proteins, have been found by [26]. The increased cholesterol-lowering effect of the proteins treated may also be linked to the structural changes. Pruning the surface hydrophobicity, along with the controlled unfolding and aggregation, can enhance protein digestibility and promote the release of bioactive peptides during digestion. A lot of research has demonstrated that peptides obtained from structurally modified legume protein have a higher capacity to bind bile acids, inhibit cholesterol micellar solubility and stimulate the activity of LDL receptors in the liver, which enhances hypocholesterolaemic activity [27]. Furthermore, ultrasound-induced conformational changes can expose sites for enzymatic cleavage that promote the formation of peptides that are important in the regulation of cholesterol metabolism [28]. Therefore, the significant structural changes that are observed in U-FPI and UH-FPI could contribute to its ability to improve the cholesterol-lowering function to some extent, as compared to untreated FPI.

### 3.4 Functional properties

#### 3.4.1 FPI solubility

**Fig. 3:**
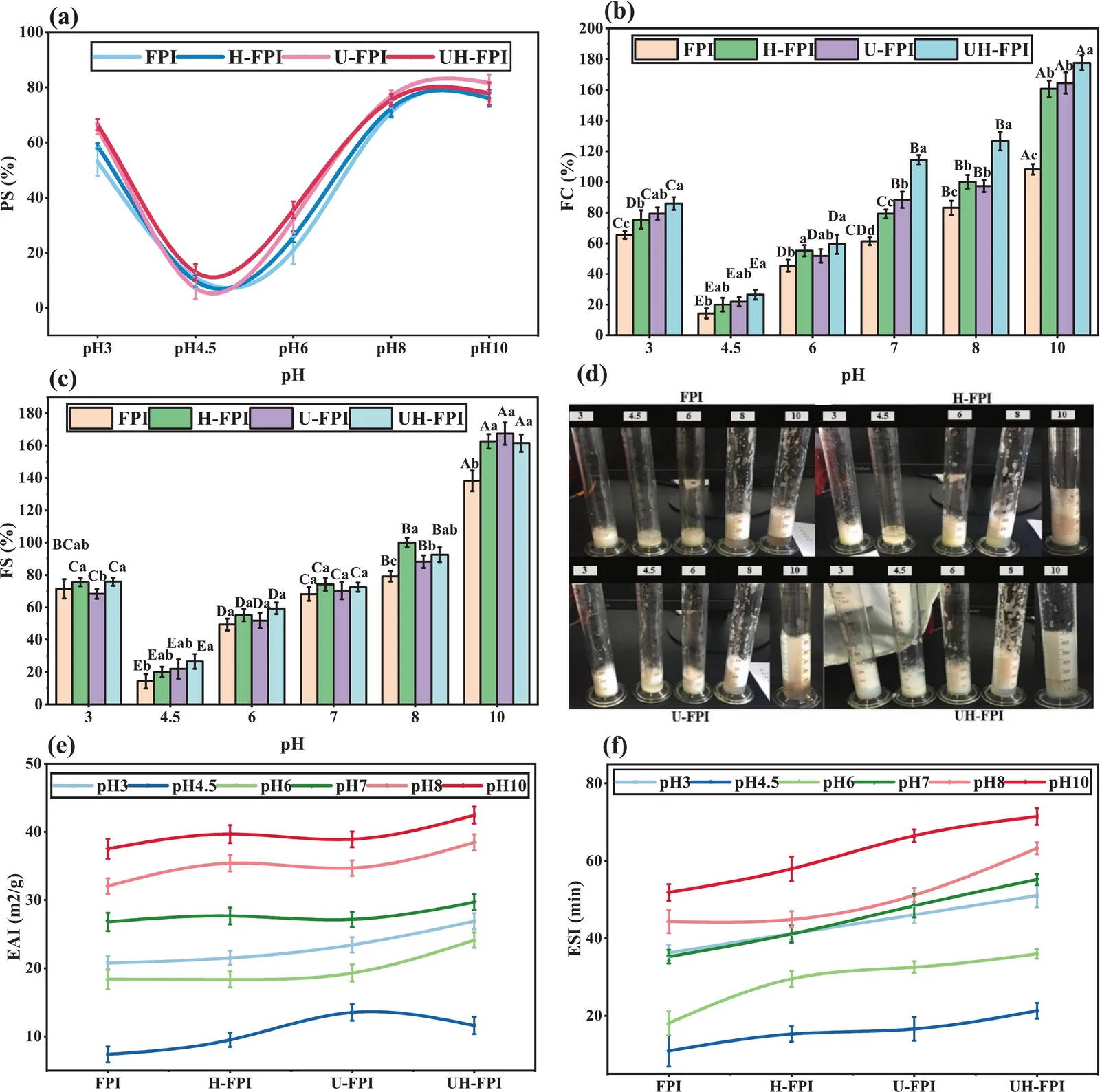
Functional attributes of FPI under different processing conditions and pH. Protein solubility (a); foaming capacity and stability (b-d) and emulsion activity index and stability (e-f) of FPI, H-FPI, U-FPI and UF-FPI.

The protein dispersibility of untreated and modified faba bean protein isolates were investigated in a pH range from 3.0 to 10.0 to assess the effect of the different treatments on the protein dispersibility, as shown in Fig. 3 (a). The solubility curve of all the samples showed the typical U-shape of legume proteins, with the minimum solubility occurring around the isoelectric point (pH 4.5). FPI had the lowest solubility at pH 3.0 (59%), while H-FPI, U-FPI and UH-FPI exhibited higher solubility values (∼67%) due to the improvement of the protein-water interactions. However, at pH 4.5, the solubility of all the treatments dropped significantly to 12–15%, suggesting that the protein's isoelectric properties were not significantly modified by the applied treatments. The solubility of the proteins under alkaline conditions (pH 8.0–10.0) was considerably higher for all the modified proteins as compared to the control, with the highest value obtained for UH-FPI (∼81%).

Structural changes caused by cavitation and controlled thermal unfolding may contribute to increased solubility after ultrasound and thermosonication. Ultrasonic cavitation breaks intermolecular associations and decreases the particle size, which provides a higher exposure of ionizable and hydrophilic groups that facilitate hydration and protein dispersion. Partial unfolding and flexibility of the molecule are also promoted with mild heat treatment without causing significant denaturation [29]. The combined thermosonication treatment would seem to induce a more balanced conformational rearrangement, which would lead to better electrostatic repulsion and less protein aggregation, especially at alkaline pH. In recent studies, similar effects of increased solubility in ultrasonically modified plant proteins have been reported, and disruption of non-covalent interactions was found to increase the molecular flexibility and water-binding ability [30]. An increase in solubility is also linked closely to an increase in bioaccessibility and functional performance of proteins during digestion. The dispersion of modified proteins may promote enzymatic hydrolysis and release of bioactive peptides with bile acid binding and cholesterol metabolism regulation effects, which may be due to increased hydration. Hence, the improved solubility of UH-FPI in the gastrointestinal tract, especially at neutral and alkaline pH, might help increase the gastrointestinal digestibility and peptide availability and contribute to the enhanced hypocholesterolaemic potential of UH-FPI.

#### 3.4.2 Foaming and emulsifying properties of FPI

The foaming properties showed a significant increase under neutral and alkaline conditions, as shown in Fig. 3 (b-d). All samples had the lowest FC (18–24%) and FS (18–28%) at pH 4.5 near the isoelectric point. Protein aggregation and a decrease in protein solubility near the isoelectric point may have also helped to decrease protein diffusion and film formation at the air–water interface. Similar decreases in foaming properties at the isoelectric pH have been reported for ultrasonically treated legume proteins [20]. At pH 6.0, the FC value rose to 48–56% while the FS value ranged from 56 to 72%, with the best stability being shown by UH-FPI. The most significant improvement was found at pH 8.0, with UH-FPI showing FC of 92% and FS of 108%, while untreated FPI showed 76% FC and 78% FS. Improved functionality of thermosonicated proteins has been explained by the changes in structure that result from cavitation and controlled thermal unfolding. Both mild heating and ultrasonic treatment degrade large aggregates of protein, decrease particle size and expose the flexible surface-active groups. The conformational changes allow protein molecules to rapidly adsorb and rearrange at the air–water interface to form cohesive and viscoelastic films that resist the coalescence and collapse of bubbles. Similar studies with faba bean and seed proteins revealed that ultrasound and thermosonication processes showed a positive effect on foaming properties due to a rise in molecular flexibility and interfacial activity, while a drop in aggregation [31].

The emulsifying properties of native and modified faba bean protein isolates were greatly influenced by pH and processing treatment, as shown in Fig. 3 (e-f). Emulsion activity index (EAI) and emulsion stability index (ESI) tended to improve after the heat, ultrasound and thermosonication treatments, but the improvement was not significant over the entire pH range. The EAI of FPI under acidic conditions (pH 3.0) was the lowest, while H-FPI, U-FPI and UH-FPI had progressively higher EAI values of 9.5 m2/g, 14.5 m2/g and 11.5 m2/g, respectively. Similarly, emulsion stability followed the same trend, and UH-FPI was found to be the most stable emulsion with ESI (21 min). The poor stability is noticed around the isoelectric point, which is due to the decreased net surface charge that results in increased protein aggregation and lowers electrostatic repulsion between emulsion droplets. These indications are in line with the earlier reported findings of [32] who observed the pH-dependent decrease in emulsifying properties in the quinoa and rice proteins, where aggregation at the isoelectric point made the protein more difficult to form an interfacial film and more likely to cause droplet flocculation.

The emulsifying properties of thermosonicated protein were better than those of the individual treatments at neutral and alkaline pH. UH-FPI exhibited the highest EAI and ESI at pH 6.0 and 7.0, increasing further at higher pH values (8.0 and 10.0) to 36.5 m^2^/g and 42.5 m^2^/g, respectively. The improvement in emulsifying properties of UH-FPI is correlated with structural modifications caused by acoustic cavitation and controlled thermal unfolding. The ultrasonication leads to the aggregate size reduction and exposes hydrophobic residues that contribute a vital part in adsorption at the oil–water interface; the mild heating results in an increase in the flexibility of molecules, but does not cause significant denaturation, which facilitates rearrangement at the oil–water interface. Similar enhancements in EAI and ESI after ultrasound-assisted thermal processing have also been recently reported for soybean, quinoa, and rice proteins [33].

### 3.5 FPI enzymatic hydrolysis (DH)

As shown in Fig. S1, the DH of faba bean protein isolate was gradually improved from 8.02% (FPI) to 12.38% (H‑FPI), 18.74% (U‑FPI), and 21.72% (UH‑FPI), showing that there are treatment-dependent increases in proteolytic susceptibility. The thermal pretreatment increased DH compared with control, due to the heat-induced partial unfolding of globular proteins, allowing previously buried endopeptidase cleavage sites to be exposed. This is similar to [34] who reported that the increasing DH was associated with pre-heat treatment above 65 °C, which resulted in higher enzyme accessibility after the proteins were unfolded. Ultrasound treatment alone yielded a more pronounced increase (DH ≈ 18.74%), consistent with acoustic cavitation-generated shear forces and microstreaming that disrupt non‑covalent interactions, thereby improving enzyme accessibility without covalently modifying the primary structure. Dual-frequency ultrasonic treatment of sunflower meal protein also showed an improvement in DH by 7.73–11.22% compared with the control and concluded that sonication induced unfolding of proteins, leading to an increase in surface hydrophobicity and altered UV–Vis spectra [35]. More importantly, thermosonication (UH-FPI) gave the highest DH (21.72%), which indicated that there was a synergistic effect between the denaturation process and ultrasonic disruption to achieve a maximum interaction between the substrate and enzyme. The studies on white kidney bean and mung bean protein have shown that the thermosonication induced significant changes in the secondary structure and tertiary structure of protein, and the maximum change in the structure was found when the thermosonication was carried out under combined conditions [17].

### 3.6 In-vitro cholesterol micellar activity

**Fig. 4:**
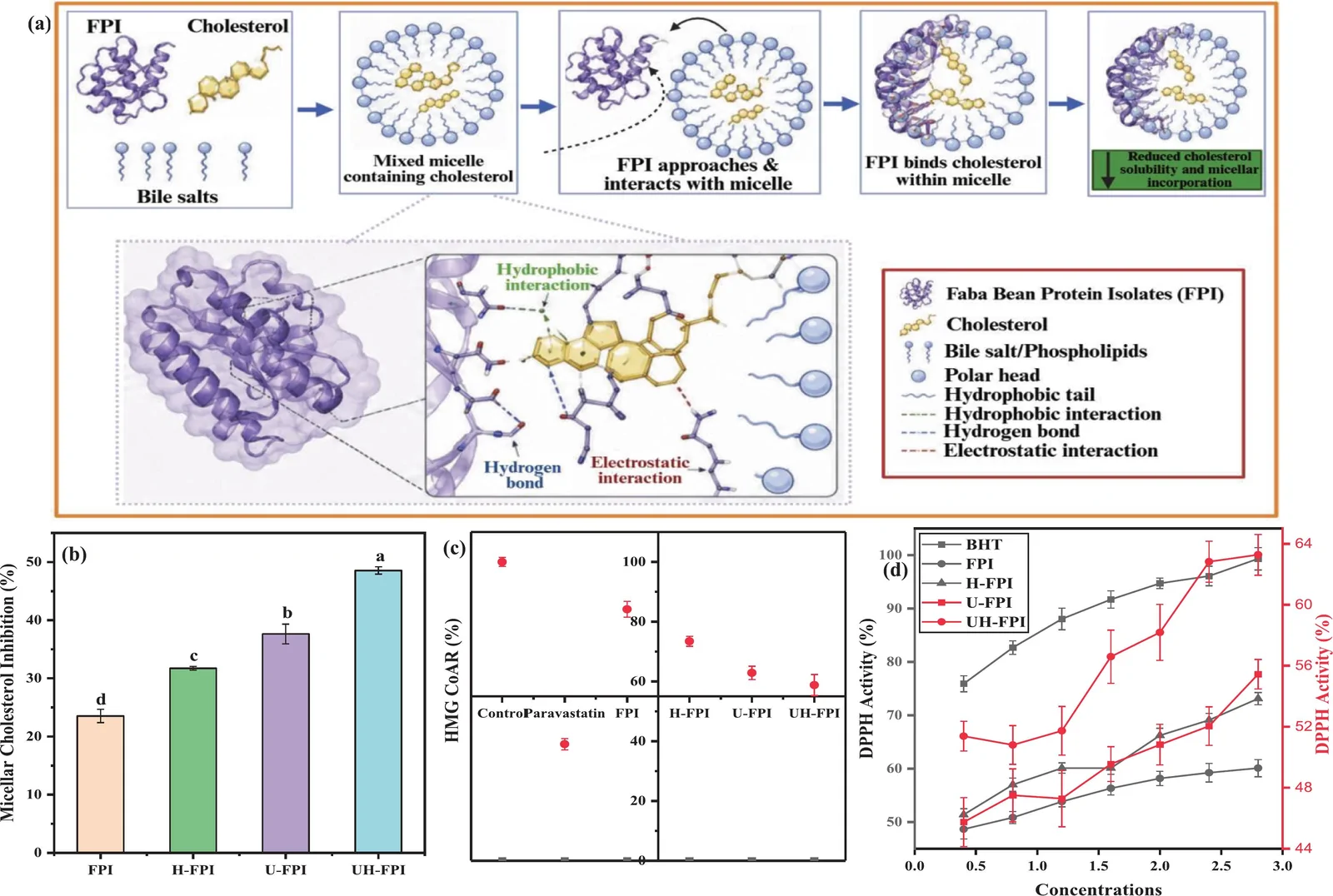
Schematic illustration of the possible mechanism of action of FPI to act and bind with cholesterol molecules within a micelle (a); In-vitro cholesterol micellar solubilization (b) and HMG CoAR activity (c); antioxidant activity (d) of FPI, H-FPI, U-FPI and UF-FPI.

The incorporation of cholesterol into bile salts, phospholipids and other lipids in the gastrointestinal tract is also important for cholesterol absorption. Thus, an in vitro cholesterol micelle model is extensively used to assess the cholesterol-lowering activity of bioactive compounds, as this closely mimics the physicochemical conditions of the human intestine, including pH profile. Cholesterol-lowering drugs can affect cholesterol absorption by altering the structure of mixed micelles, competing with cholesterol to enter the micelle, enlarging micelle size, and interacting with the micellar membrane, decreasing the solubilization of cholesterol and subsequent intestinal uptake [15], [17], [36]. The proposed mechanism for cholesterol incorporation into mixed micelles and the inhibitory effect of structurally modified FPI on micellar cholesterol incorporation are shown in Fig. 4 (a).

The inhibition efficiency of the native FPI was the lowest (23.0%), while that of H-FPI, U-FPI and UH-FPI was progressively increased (32.0%, 37.0% and 48.0%, respectively) as exhibited in Fig. 4 (b). The significant improvement in cholesterol binding capacity after the thermosonication treatment clearly shows the effect of the combined ultrasound and heat treatment. This improvement can be correlated with the structural changes that occur during the processing, such as partial unfolding, breakage of intermolecular aggregates and changes in the surface hydrophobicity. Conformational changes may promote increased exposure of mixed micelles and cholesterol-binding hydrophobic amino acid residues during gastrointestinal digestion. Under the influence of cavitation, a high-speed shear effect is created, causing the proteins to unfold and generate smaller peptides, and at the same time, a transient high-energy microenvironment is created due to cavitation, which causes the proteins to become flexible and enables controlled heating without denaturation. The two effects in combination may have made non-polar regions more accessible for interaction with cholesterol molecules. In previous studies, structural modification of legume and plant proteins by physical processing has been shown to increase the surface activity and conformational flexibility, which leads to enhanced bile acid binding, cholesterol micellar disruption and lipid adsorption capacity. According to [15], [17] the hypocholesterolaemic functionality of the plant protein has been improved through a modification process using ultrasound, which involves improving its hydrophobic interactions and interfacial behavior. Likewise, [17] UH treatment caused structural rearrangements that led to better lipid binding properties. The synergistic effect of protein unfolding and aggregation observed in the thermosonication process resulted in superior cholesterol inhibition, implying a more favorable balance between these two processes as a result of the combined effect compared to individual protein unfolding or aggregation. Better micellar cholesterol inhibition could also result in a decrease in intestinal cholesterol absorption, as the ability to solubilize cholesterol and enter the mixed micelle is reduced. In this regard, thermosonication seems to be an efficient non-chemical processing method for enhancing the bioactive functionality of faba bean proteins and the techno-functional properties of the proteins for the production of functional food ingredients.

We also performed a correlation analysis between secondary structure indicators and Ho, DH, and cholesterol micellar activity, as shown in Table 1. Among significant correlations, A-P was negatively correlated with hydrophobicity (−0.986) and DH (−0.955); similarly, α-helix was also negatively linked with β-inter (−0.964) and β-intra (−0.989) sheets. On the other hand, β-inter was directly linked with β-intra (0.989), likewise, surface hydrophobicity was positively associated with the DH (0.983).

**Table 1:** Pearson correlations between secondary structure indicators and Ho, DH, and cholesterol micellar activity.

	**β-turn**	**α-helix**	**R.C**	**β-inter**	**β-intra**	**Ho**	**DH**	**Cholesterol**
**A-P**	0.832	0.683	−0.636	−0.852	−0.771	−0.986*	−0.955*	0.901
**β-turn**		0.222	−0.932	−0.466	−0.36	−0.738	−0.63	0.644
**α-helix**			0.118	−0.964*	−0.989*	−0.796	−0.826	0.587
**R.C**				0.149	0.021	0.498	0.399	−0.534
**β-inter**					0.989*	0.927	0.934	−0.74
**β-intra**						0.865	0.875	−0.642
**Ho**							0.983*	−0.887
**DH**								−0.929

Asterisks * on each correlation indicate significance at 0.05.

### 3.7 HMG CoA activity

Processing treatment significantly affected the inhibitory activity of native and modified FPI’s against 3-hydroxy-3-methylglutaryl coenzyme A reductase (HMG-CoAR). The enzymatic activity of the positive control (pravastatin) remained at 98% of the reference activity, while the enzyme activity decreased gradually in the presence of treated protein samples. Fig. 4 (c) illustrates that H-FPI, U-FPI, and UH-FPI had residual HMG-CoAR activities of 75%, 65%, and 60%, respectively, which represents 15%, 25%, and 40% inhibition. These results suggest that thermosonication was more effective in improving the inhibitory properties of FPI than either heat treatment or ultrasound treatment. The enhanced inhibition seen following a thermosonication process could relate to changes in structure that occur as a result of both ultrasonic cavitation and controlled heating. The conformational changes could enhance the accessibility to enzymes and allow the release or exposure of HMG-CoAR-affinity bioactive peptide sequences. It has been reported that ultrasound-assisted processing can lead to the formation of low molecular weight peptides and to greater bioactivity of legume proteins due to the disruption of non-covalent interactions and the increase in protein susceptibility to hydrolysis. The study by [17] showed that thermosonication significantly changes the structural properties and bioactivity of mung bean and kidney bean proteins and increases their functionality for cholesterol. The larger inhibitory effect of UH-FPI also indicates that thermosonication can either facilitate the discovery or exposure of peptide regions that interact with the catalytic domain of HMG-CoAR. Food-derived peptides have been reported to inhibit HMG-CoAR by competitive or mixed inhibition depending on the size, hydrophobicity and amino acid composition of the peptide. [37] pointed out that peptides with low molecular weight and hydrophobic properties have improved affinity for cholesterol-regulating enzymes. In a similar study, [38] found that after simulated gastrointestinal digestion, peptides with significant HMG-CoA reductase inhibitory activity were produced from faba bean proteins. The limitation of the present study was the absence of a protein matrix blank control in the HMG-CoA reductase inhibition assay. Future studies should include such a control to further validate the enzyme inhibition due to the bioactive material of modified FPI rather than the matrix or interference effects. Overall, these results suggest that thermosonication is a promising alternative green processing method for enhancing the hypocholesterolaemic activities of faba bean protein ingredients for nutraceutical applications and functional foods.

### 3.8 Antioxidant activity

The DPPH radical scavenging activity of native and modified FPI increased gradually with increasing protein concentration (from 0.07 to 3.0 mg/mL). As shown in Fig. 4 (d), at the lowest concentration (0.07 mg/mL), the antioxidant activity varied between moderate (44%–50%), and there were only small differences between the treatments. At higher concentrations, however, there were substantial variations. At the concentration of 3.0 mg/mL, native FPI had 70% scavenging activity, while H-FPI had moderate activity of up to 74%. The ultrasound-treated (U-FPI) and thermosonicated (UH-FPI) samples, however, showed 100% radical scavenging activity at the highest concentration, higher than the synthetic antioxidant, BHT (99%). Furthermore, the combined treatment (UH-FPI) required a lower concentration than ultrasound alone (U-FPI) for maximum inhibition, showing that the synergistic effect of the combined treatment was greater than that of ultrasound alone. Ultrasonic and thermosonication treatments were observed to cause structural changes that increased the antioxidant activity, which might be related to cavitation and controlled thermal unfolding [17], [35].

The local shear forces and transient cavitation from high-intensity ultrasound cause intermolecular interactions to be disrupted and partially unfold protein structures. Conformational changes can expose more amino acid residues that have hydrogen-donating and electron-transfer capabilities, such as tyrosine, tryptophan, phenylalanine, cysteine, and histidine [39]. These residues can be made more accessible in proteins and their derived peptides, thus increasing the potential to neutralize free radicals. Some of the same antioxidant benefits have been observed with legume and seed proteins after ultrasound-assisted processing, as the structural unfolding and release of peptides resulted in improved DPPH radical scavenging ability. The antioxidant activity of legume proteins was enhanced by conformational modification and making more reactive amino acid residues available for reaction, as shown by [17]. Similarly, [38] observed that physical processing technologies create and liberate antioxidant peptides with greater radical scavenging activity.

### 3.9 In-vivo activities

#### 3.9.1 Feed intake and body weight

Over the 28‑day feeding period, body weight changes were significantly (*p* < 0.05) different among dietary groups (Table 2), suggesting that protein supplementation reduces the weight gain of high‑fat (HF) diet-fed hamsters. There was baseline variability in the initial body weights, with the highest (HF group; 20.76 ± 4.56  g) and lowest (UH‑FPI group; 13.15 ± 3.67  g) values, but these were not significant at Day 7. The HF group had continued weight gain by Day 14 (24.28 ± 5.64  g), similar to that of an obesogenic high dietary fat. High-fat diet supplementation with either soy protein isolate (SPI) or thermosonicated faba bean protein isolate (UH‑FPI) led to reduced weight gain (17.48 ± 8.40  g and 21.10 ± 5.67  g, respectively), indicating that higher protein intake may be a countermeasure to the high-fat-induced weight gain. Both protein supplements showed significant body weight gains over time, with the highest body weight gain being achieved by the HF group (20.57 ± 4.07  g), the lowest by the SPI group (7.37 ± 4.00  g) and the UH‑FPI group (10.65 ± 6.69  g).

**Table 2:** Growth parameters of hamsters fed a high-fat diet. Means ± standard errors (SE) were determined for 10 hamsters per group. Different superscript letters indicate significant differences at *P* < 0.05. Body weight (BW).

	**Initial BW (g)**	**Final BW (g)**	**BW gain (g)**	**Food Intake (g)**
**Control**	89.01 ± 3.39^A^	108.19 ± 8.98^AB^	19.18 ± 4.56^A^	6.49 ± 0.61^A^
**SPI**	89.11 ± 4.34^A^	96.48 ± 11.00^C^	7.37 ± 4.00^B^	6.51 ± 0.43^A^
**UH-FPI**	89.49 ± 3.22^A^	100.14 ± 6.69^B^	10.65 ± 6.75^B^	6.48 ± 0.33^A^
**High Fat**	90.46 ± 3.78^A^	111.03 ± 4.07^A^	20.57 ± 5.64^A^	6.50 ± 0.52^A^

The observed trend is consistent with the findings from rodent studies that raising the protein-to-carbohydrate ratio in obesogenic diets lowers weight gain and adiposity, perhaps due to greater diet-induced thermogenesis and lower feed efficiency [40]. Additionally, consuming more protein has been found to boost energy expenditure, improve satiety signalling, and cause changes in substrate oxidation, which all work to reduce net weight gain in a high-fat environment [41]. Sustained attenuation of weight gain in UH-FPI compared to HF controls may be due to improvements in protein quality and digestibility resulting from thermosonication, potentially leading to greater amino acid availability and metabolic signalling. In recent studies, plant protein–rich diets were reported to affect energy metabolism and to help to maintain lean body mass, which would result in better weight control when feeding high fat [40]. Moreover, protein supplementation can affect the gut hormones which regulate food intake (such as GLP‑1, CCK) and boost metabolic rate, leading to even more reduced gain of body weight in protein-fed groups compared to groups fed high fat [41]. These data collectively suggest that high-fat diet–induced weight gain can be prevented, and weight loss can be achieved with structured protein supplementation, such as UH-FPI, thereby providing clues for the development of nutritional strategies for obesity prevention and improvement of metabolic health.

#### 3.9.2 Serum biochemical analysis

**Fig. 5:**
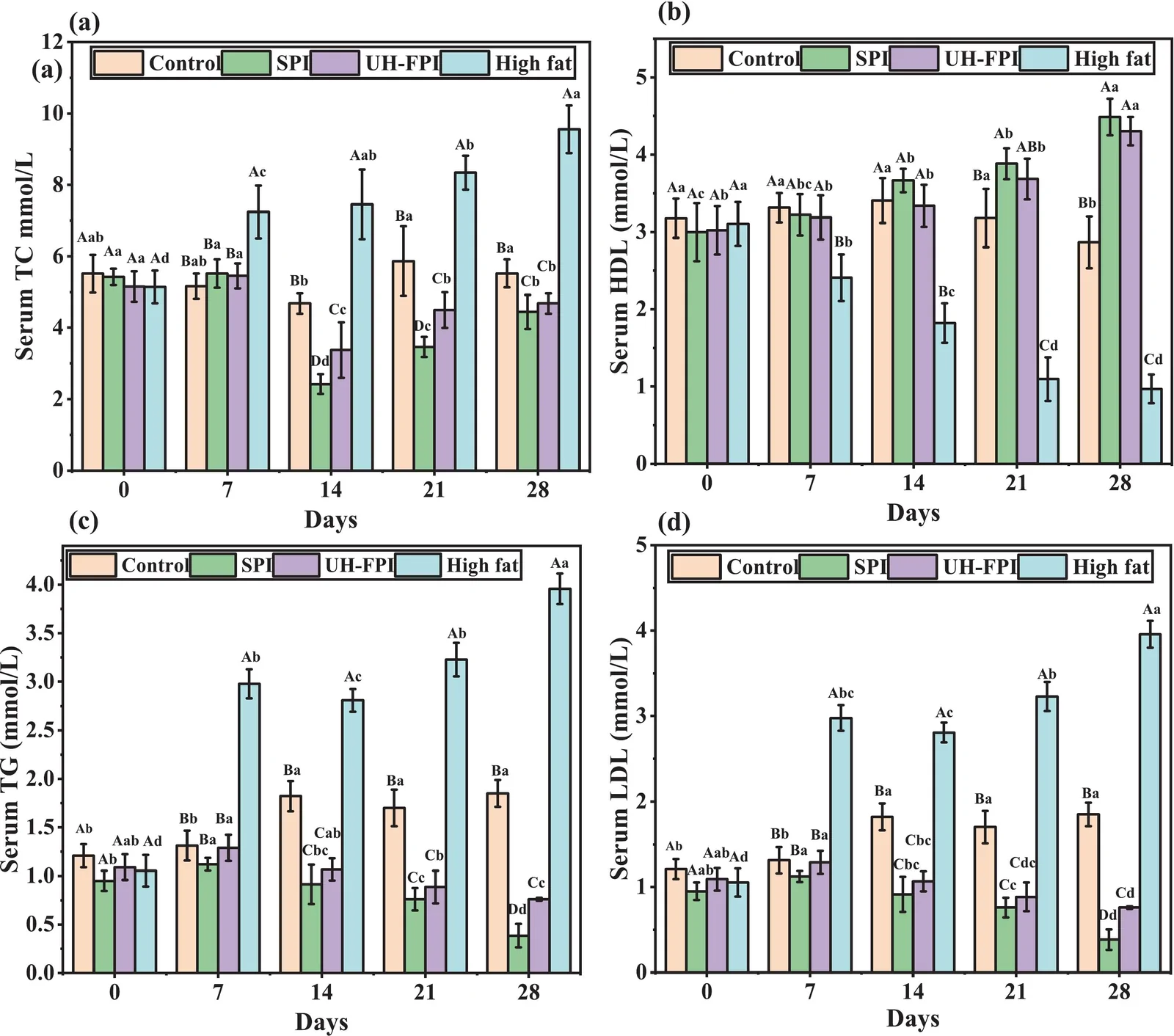
Effect of UH-FPI on serum profiles. Serum total cholesterol (a); serum HDL (b); serum TG (c), and serum LDL (d). Different letters indicate the level of significance at p < 0.05.

Hamsters were fed a high-fat diet for 28 days with dietary supplementation of native FPI, UH-FPI (thermosonicated faba bean protein isolate) and SPI (soy protein isolate), which significantly influenced the serum lipid profiles in the 28-day feeding study. During the experimental period, the levels of total cholesterol (TC) significantly (*p* < 0.05) rose in the high-fat control group from 5.1 to 9.5 mM, triglycerides (TG) from 1.00 to 4.00 mM, and LDL cholesterol from 1.2 to 4.0 mM, while HDL remained relatively low, as shown in Fig. 5 (a-d). On the other hand, dietary supplementation with SPI alone had beneficial effects on lipid metabolism, leading to decreased TC, TG and LDL, and increased HDL levels. In the UH-FPI group, TC was not significantly affected (5.2–4.8 mM), TG was significantly reduced (p < 0.05) (1.00–0.40 mM), LDL cholesterol was reduced (1.0–0.8 mM), and HDL was increased (3.0–4.2 mM). The results showed that the lipid-lowering activity of UH-FPI was similar to or higher than that of SPI. A beneficial serum lipid profile achieved in the UH-FPI group could be tightly linked to the superior in vitro inhibition capacity of micellar cholesterol. The incorporation of micellar cholesterol was inhibited by 48% by UH-FPI, which is much higher than native FPI (23%) and ultrasound-treated protein (37%). This increased inhibition might inhibit the absorption of cholesterol in the intestine by blocking the incorporation of cholesterol into mixed micelles and enhancing the removal of bile acids, leading to a decrease in serum TC and LDL levels [42]. Thermosonication may also be responsible for the superior hypolipidemic activity of UH-FPI, due to structural modifications. These results from far-UV CD and surface hydrophobicity are consistent with the effect of combined ultrasound and mild heat treatment, which disrupts intermolecular aggregates, promotes partial unfolding and enhances exposure of hydrophobic regions, which are involved in cholesterol and bile acid interactions. The same was observed by [30] who showed that thermosonication is an effective method for improving functional and bioactive properties of plant proteins by conformational rearrangements and increased surface activity. Partial unfolding during thermosonication can also help release the bioactive peptides that inhibit HMG-CoA reductase activity, which can affect endogenous cholesterol biosynthesis. Similarly, plant proteins have been reported to be comparable hypocholesterolaemic agents in hypercholesterolemic hamsters, which were correlated with modulation of bile acid metabolism and intestinal lipid absorption, by [43]. In conclusion, the results indicated that the combined effect of thermosonication on both cholesterol absorption and lipid metabolism could yield a more efficient effect on FPI in the cholesterol-lowering activity.

#### 3.9.3 Determination of liver and intestinal lipid metabolism biomarkers

**Fig. 6:**
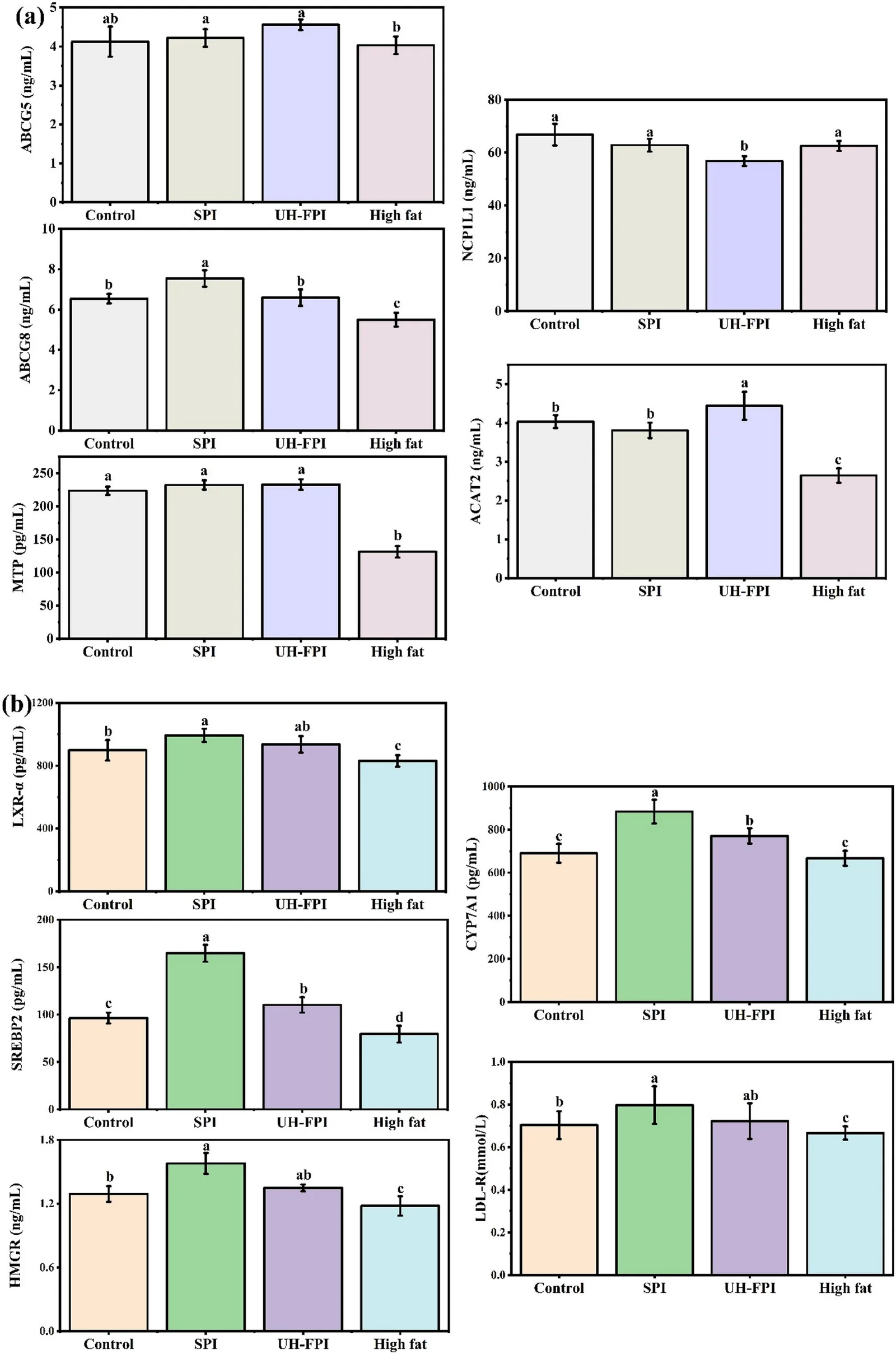
Thermosonicated faba bean protein isolate (UH-FPI) influence on the expression of cholesterol metabolism proteins in hamsters fed a high-fat diet. Hepatic cholesterol metabolism (a) and Intestinal cholesterol transportation (b).

To better understand the molecular mechanism underlying the cholesterol-lowering activity of UH-FPI, the expression levels of various proteins associated with intestinal cholesterol transport and hepatic cholesterol metabolism were examined. Absolute protein concentrations are shown in Fig. 6, and HF-normalized relative protein expression changes are shown in Supplementary Figs. S2- S3 to illustrate the degree of regulation induced by UH-FPI. Both analyses revealed that UH-FPI was able to affect several different targets related to cholesterol, indicating a coordinated regulation of cholesterol availability in the intestine and hepatic cholesterol homeostasis.

##### 3.9.3.1 The regulation of intestinal cholesterol absorption and efflux

The intestine is a major site where dietary cholesterol absorptive capacity is regulated and is a key target for dietary interventions to combat hypercholesterolemia. UH-FPI supplementation caused a significant (P < 0.05) increase in expression of ABCG5 and ABCG8, and a significant (P < 0.05) decrease in expression of NPC1L1, compared with the HF group Fig. 6 (a). These regulatory effects were also verified by the HF-normalized analysis (Fig. S2). NPC1L1 is the major intestinal cholesterol transporter that transports dietary and biliary cholesterol into enterocytes, while ABCG5/ABCG8 transports excess cholesterol from enterocytes back into the intestinal lumen, thereby reducing the net absorption of cholesterol [8]. ABCG5 and ABCG8 are heterodimeric ATP-binding cassette transporters that promote the reverse movement of cholesterol from enterocytes into the intestinal lumen and thus reduce the net uptake of cholesterol. Concurrently, both the down-regulation of NPC1L1 and the up-regulation of ABCG5/ABCG8 indicate that UH-FPI decreases cholesterol availability in the intestine by the combined action of cholesterol uptake and efflux. Plant peptides and functional foods with hypocholesterolemic activity have been reported to have similar mechanisms [44]. The molecular changes are similar to the increase in cholesterol micellar solubility inhibition in the in vitro assay, suggesting that UH-FPI might have a dual effect on cholesterol absorption, physicochemical interaction, as well as biological modulation of intestinal cholesterol transport mechanisms. Previous research has shown that peptides derived from plant proteins with both hydrophobic and charged amino acids can bind cholesterol and bile salts and inhibit cholesterol micellar solubilisation and intestinal uptake [44]. Thus, the cholesterol-lowering activity of UH-FPI could be due to a physicochemical inhibition of cholesterol availability as well as to a biological regulation of intestinal cholesterol transport.

Interestingly, the UH-FPI group had a higher expression of ACAT2 and MTP than the HF group. ACAT2 is involved in intercellular cholesterol esterification, while MTP controls the assembly and secretion of apoB-containing lipoproteins. The upregulation of these proteins may be a restoration of normal enterocyte lipid metabolism in response to reduced cholesterol overload, rather than increased cholesterol absorption. This interpretation is in line with the proposed function of SREBP2 and HMGCR in cholesterol regulation in the cell by a sterol-dependent feedback mechanism [45]. A similar compensatory regulation of lipid transport-related proteins has been reported when recovering from metabolic imbalance induced by a high-fat diet [46].

##### 3.9.3.2 The regulation of hepatic cholesterol synthesis and elimination

The liver regulates cholesterol balance in the body in a coordinated way by controlling cholesterol uptake, endogenous cholesterol production, conversion to bile acids and elimination. UH-FPI significantly (P < 0.05) affected proteins involved in liver cholesterol metabolism, including LXR-α, CYP7A1, LDL-R, SREBP2, and HMGCR (Fig. 6 (b). This was further revealed by the normalized expression analysis, which showed the relative extent of UH-FPI-mediated regulation compared to the HF group (Fig. S3). The nuclear receptor LXR-α is a critical cholesterol-sensitive receptor involved in cholesterol efflux and bile acid metabolism. LXR-α is activated by inducing CYP7A1 expression, the rate-limiting enzyme in the conversion of cholesterol to bile acids. Therefore, the increased expression of LXR-α and CYP7A1 after UH-FPI supplementation suggests enhanced hepatic cholesterol conversion and elimination through bile acid synthesis. The regulation of the LXR-α/CYP7A1 pathway has been known as one of the key pathways involved in dietary control of cholesterol metabolism [46], [47]. Furthermore, UH-FPI stimulated LDL-R expression, suggesting improved clearance of circulating LDL cholesterol from the liver. The uptake of plasma LDL via LDL-R is a primary mechanism of regulating circulating cholesterol levels, and dietary bioactive peptides have been shown to modulate LDL metabolism by regulating cholesterol homeostasis in the liver [48].

SREBP2 is an important transcription factor involved in the regulation of cholesterol biosynthesis that regulates the expression of HMGCR, which is a rate-limiting enzyme involved in endogenous cholesterol synthesis. However, the expression of UH-FPI compared with the HF group was higher, but the change in expression should not be considered an improvement in cholesterol synthesis. In hyperlipidemic conditions, the excess cholesterol blocks the normal cholesterol sensing and feedback regulation within the cell. Thus, the observed increase in SREBP2/HMGCR expression after UH-FPI treatment might represent a recovery of physiological cholesterol homeostasis, rather than an increase in cholesterol biosynthesis. This interpretation is supported by the actions of SREBP2 and HMGCR, which are involved in the regulation of intracellular cholesterol levels by sterol-dependent feedback mechanisms [45]. Collectively, these alterations indicate that UH-FPI improves hepatic cholesterol homeostasis by promoting cholesterol clearance through LDL uptake and bile acid synthesis while regulating endogenous cholesterol biosynthesis.

##### 3.9.3.3 Integrated mechanism of UH-FPI-mediated cholesterol regulation

In combination, UH-FPI modulated cholesterol metabolism by two parallel pathways. UH-FPI inhibited cholesterol absorption in the intestine by inhibiting NPC1L1 cholesterol uptake and by promoting ABCG5/ABCG8 cholesterol efflux. UH-FPI restored intracellular cholesterol regulatory mechanisms in the liver by enhancing LDL-R-mediated cholesterol clearance and activation of the LXR-α/CYP7A1 pathway for bile acid synthesis, thereby promoting cholesterol disposal. The enhanced cholesterol-regulating activity of UH-FPI may be attributed to thermosonication-induced structural remodeling of faba bean proteins. Ultrasound-assisted modification can disrupt non-covalent bonds, change protein conformation, expose functional groups, enhance enzymatic access to protein surfaces, and help to produce bioactive peptides during digestion. Recent research has shown that plant protein treated with ultrasound can be more effective at lowering cholesterol levels than untreated proteins because of increased peptide release and increased exposure of cholesterol interaction sequences [49]. Thus, the multi-target regulation following UH-FPI supplementation could be due to the combined effects of thermosonication-induced structural modification, improved peptide bioavailability and modulation of cholesterol metabolic pathways.

## 4 Conclusion

The present study showed that the heat treatment, ultrasonication, and sequential thermosonication significantly affected the structural and functional properties of FPI, with the highest degree of structural remodeling observed in UH-FPI. The conformational changes induced by thermosonication were confirmed by FTIR and CD analyses, indicating alterations in protein secondary structure and molecular interactions. These structural changes improved the exposure of functional groups and enzymatic accessibility, resulting in enhanced techno-functional properties such as protein solubility, foaming capacity and stability, emulsifying activity and stability. Furthermore, the increased antioxidant activity and cholesterol micellar inhibition of UH-FPI indicated that thermosonication enhanced the biological potential of FPI. The cholesterol-lowering effect of UH-FPI was also confirmed in HF diet hamsters. The expression of proteins related to cholesterol transport, absorption, biosynthesis and bile acid metabolism was shown to be modulated significantly by UH-FPI. The relative change in concentration of these different molecular targets was further illustrated by the HF-normalized expression profiles. UH-FPI controlled cholesterol metabolism in two different complementary mechanistic pathways. UH-FPI inhibited cholesterol absorption in the intestine by down-regulating NPC1L1 expression and increasing the ability of ABCG5/ABCG8 to promote cholesterol efflux. In the liver, UH-FPI enhanced cholesterol homeostasis by regulating LDL-R-mediated cholesterol removal and encouraging cholesterol conversion to bile acid via the LXR-α/CYP7A1 pathway. The results suggest that UH-FPI has cholesterol-lowering properties via synergistic modulation of intestinal cholesterol transport and hepatic cholesterol metabolism.

The overall results clearly support the link between the thermosonicated structure and hypocholesterolemic functionality of FPI. UH-FPI is therefore a good functional ingredient for the formulation of cholesterol-lowering foods and nutraceuticals to promote cardiovascular health. Future work should be directed towards investigating the gastrointestinal fate of UH-FPI, the release and bioavailability of bioactive peptides, and the comprehensive safety assessment of UH-FPI as a food ingredient for health promotion.

## CRediT authorship contribution statement

**Jawad Ashraf:** Writing – original draft, Supervision, Software, Methodology, Investigation, Formal analysis, Conceptualization. **Muawuz Ijaz:** Writing – review & editing, Software, Data curation. **Zeeshan Ali:** Visualization, Validation. **Sanabil Yaqoob:** Visualization, Data curation. **Miao Bai:** . **Hafiz Umer Javeed:** Visualization, Data curation. **Ayhsa Imtiaz:** Visualization, Formal analysis. **Sumei Zhou:** . **Qing Shen:** Writing – review & editing, Methodology, Data curation. **Ting Chen:** Writing – review & editing, Resources, Funding acquisition.

## Declaration of competing interest

The authors declare that they have no known competing financial interests or personal relationships that could have appeared to influence the work reported in this paper.

## Appendix A Supplementary data

Supplementary Data 1 The supplementary material provides additional information supporting the findings presented in this study.
