# Supplementary material for: Thermosonication-induced structural remodeling of faba bean protein: Improved functionality, digestibility, and cholesterol metabolism regulation for cardiovascular health
Source: Ultrason Sonochem. 2026 Sep 4;133:108038. doi: 10.1016/j.ultsonch.2026.108038 (PMC13586586; doi:10.1016/j.ultsonch.2026.108038)
Supplement: Supplementary file 1 [file mmc1.docx]

**Supplementary**

**Thermosonication-induced structural remodeling of faba bean protein: Improved functionality, digestibility, and cholesterol metabolism regulation for cardiovascular health**

Jawad Ashraf ^a,*^, Muawuz Ijaz ^b^, Zeeshan Ali ^a^, Sanabil Yaqoob^c^, Bai Miao ^a^, Hafiz Umer Javeed ^d^, Ayhsa Imtiaz^e^, Zhou Sumei ^f,*^, Qing Shen^c,*^, Ting Chen^g,*^

*^a^ College of Food Science and Engineering, Bohai University, Jinzhou, Liaoning 121013, China.*

*^b^ Department of Animal Sciences, CVAS-Jhang 35200, University of Veterinary and Animal Sciences, Lahore 54000, Pakistan.*

*^c^ Laboratory of Food Nutrition and Clinical Research, Institute of Seafood, Zhejiang Gongshang University, Hangzhou, 310012, China*

*^d^ Guangxi Zhuang Autonomous Region Engineering Research Centre of Marine Food Nutrition and Processing Technology Innovation, Guangxi College and University Key Laboratory of High-Value Utilization of Seafood and Prepared Food in Beibu Gulf, College of Food Engineering, Beibu Gulf University, Qinzhou, 531011 China.*

*^e^ School of Fisheries and Life Sciences, Shanghai Ocean University, Shanghai, China*

*^f^ School of Food and Health, Beijing Technology and Business University, Beijing 100048, China.*

*^g^ Department of Cardiology, First Affiliated Hospital, Zhejiang University School of Medicine, Hangzhou, 310003, China*

**Table S1** Ingredients proportion (g/100 g diet) of experimental diets^1^

| **Ingredients** | **Control** | **High fat** | **SPI** | **UH-FPI** |
| --- | --- | --- | --- | --- |
| Protein | 20 | - | 20 | 20 |
| Corn Starch | 40 | 40 | 40 | 40 |
| Soybean Oil | 7 | 7 | 7 | 7 |
| Cellulose | 5 | 5 | 5 | 5 |
| Sucrose | 10 | 10 | 4 | 4 |
| L-Cystine | 0.30 | 0.30 | 0.30 | 0.30 |
| Maltodextrin 10 | 13 | 13 | 13 | 13 |
| t-Butylhydroquinone | 0.0015 | 0.0015 | 0.0015 | 0.0015 |
| Mineral Mix | 3.50 | 3.50 | 3.50 | 3.50 |
| Vitamin Mix | 1 | 1 | 1 | 1 |
| Choline Bitartrate | 0.25 | 0.25 | 0.25 | 0.25 |
| Cholesterol | 1 | 1 | 1 | 1 |
| Fat | 0 | 5 | 5 | 5 |
| Biliary Salt | 0.21 | 0.21 | 0.21 | 0.21 |

^1^According to AIN-93G formula.

**Table S2:** Amino acid profile of Faba bean protein in mg/g.

|  | **FPI** |
| --- | --- |
| **Asp** | 80.35 ± 3.81 |
| **Thr** | 24.69 ± 1.27 |
| **Ser** | 36.38 ± 1.77 |
| **Glu** | 143.94 ± 6.80 |
| **Gly** | 28.30 ± 1.33 |
| **Ala** | 29.21 ± 1.40 |
| **Cys** | 4.03 ± 0.12 |
| **Val** | 35.60 ± 1.55 |
| **Met** | 10.56 ± 0.36 |
| **Ile** | 34.00 ± 1.37 |
| **Leu** | 60.91 ± 2.86 |
| **Tyr** | 29.51 ± 1.15 |
| **Phe** | 35.42 ± 1.85 |
| **Lys** | 47.11 ± 2.37 |
| **His** | 18.60 ± 1.12 |
| **Arg** | 64.83 ± 4.36 |
| **Pro** | 112.75 ± 5.09 |

**Table S3:** Composition of Faba bean protein in %.

|  | **FPI Composition** |
| --- | --- |
| **Protein** | 90.58 ± 0.48 |
| **Moisture** | 2.57 ± 0.09 |
| **Ash** | 4.33 ± 0.24 |
| **Fat** | 0.85 ± 0.26 |
| **Carbohydrate** | 2.3 ± 0.13 |

**Figure S1:** Degree of hydrolysis of FPI, H-FPI, U-FPI and UH-FPI

**Figure S2: HF-normalized relative expression changes of cholesterol metabolism in the intestine.**

**Figure S3: HF-normalized relative expression changes of cholesterol metabolism in the liver.**
